# Martin Ritter: Into the world: the movement of Patočka’s phenomenology: Springer, Cham, 2019, 183 pp, ISBN 978-3-030-23656-4

**DOI:** 10.1007/s11007-020-09516-7

**Published:** 2020-11-24

**Authors:** Martin Koci

**Affiliations:** University of Vienna, Vienna, Austria

**Keywords:** Jan patočka, Phenomenology, World, Existence

## Abstract

The studies of the Czech phenomenologist Jan Patočka (1907–1977) has been flourishing recently. Martin Ritter’s book *Into the World: The Movement of Patočka’s Phenomenology* (Springer 2019) offers an important contribution to the debate and a long-awaited critical presentation of Patočka’s asubjective phenomenology as well as creative re-reading of Patočka's central doctrine of the movements of existence.

Recent years have been rich with studies of the Czech phenomenologist Jan Patočka (1907–1977). The past year alone has seen the publication of four monographs, each of which deals with Patočka’s thought from a different angle: a phenomenological reading is offered by Chiara Pesaresi,^1^ a political reading by Aspen Brinton,^2^ and a theological perspective by Martin Koci.^3^ Martin Ritter’s book offers an important contribution to the debate and a long-awaited critical presentation of Patočka’s phenomenological philosophy in English. The need to fill certain gaps in Patočka studies is reflected in the two-fold structure of Ritter’s argument: Part I provides an analytical and chronological reconstruction of Patočka’s phenomenology; Part II is a systematic exploration of “rethinking existence” with and beyond Patočka’s a-subjective phenomenology. The two parts are interrelated. Ritter suggests that the logic of Patočka’s intellectual evolution can be read as a series of tireless attempts to overcome “the subjectivist shortcomings of transcendental phenomenology” (p. 6), thus revising both Husserl’s notion of the transcendental ego and Heidegger’s *Dasein.* In this respect, Patočka struggles with a number of concepts throughout his professional career, beginning with inwardness (*nitro*) and life, later replaced by a phenomenological re-conceptualization of the Platonic Idea, and later still re-considered in relation to the world and the body, and finally arriving at a phenomenological-existentialist reinterpretation of the Aristotelian movement. In his critical-constructive engagement with Patočka, Ritter takes the movement of existence as the central and most promising part of Patočka’s phenomenological corpus as it is the concept which enables the overcoming of both subjectivism and objectivism; or, to use Ritter’s vocabulary, to materialize the intertwining between the objective and the spiritual.

The device of following Patočka from the early years of his dissertation on the notion of evidence and his habilitation on the natural world, through the project of Negative Platonism, to mature writings such as *Heretical Essays in the Philosophy of History* offers a novel perspective on reading Patočka’s apparently varied and unsystematic body of work. Ritter’s interpretation complements that of Paul Ricoeur, who finds the twin foci of Patočka in his philosophy of history and the problem of the natural world. Ritter identifies the core of Patočka’s interest in the concept of life, or existence, which leads the Czech phenomenologist into his development of an a-subjective phenomenology, in which, nonetheless, the subject is certainly still present. The question is, “How does the subject come to itself?” Patočka is satisfied, Ritter suggests, neither with Husserl’s transcendentalism nor with Heidegger’s being-thrown-in-the-world without really moving in the world and explores the interaction of the subject with a phenomenal field that is not dependent on that subject.

The first part of Ritter’s book therefore serves as an introduction not only to Patočka, something previously missing in English, but also to a-subjective phenomenology. Nonetheless, it is the second part of the book, which scrupulously elaborates on the movement of existence, which I find worthy of special attention and further debate. Patočka’s doctrine recognizes the three movements of acceptance, defence, and transcendence, the last of which functions as the fully authentic movement and the completion of the previous two and transcends the sphere of the mere preservation of life.

Ritter introduces the problem through the dialectic between the body and the soul. Against the ontogenetical reading that the body gives rise to subjectivity (as proposed by Renaud Barbaras, for example), Ritter interprets the body as the objective bearer of subjectivity, and thus gives preference to the *Körper* over the *Leib*. Along the lines of Findlay’s research on Patočka,^4^ the soul is understood metaphorically, not as a metaphysical entity but as a faculty of a responsible way of being and a life of reflection which nonetheless does not lose sight of what transcends the world. At this point, Ritter takes issue with Patočka, whom he sees as an irredeemable transcendentalist who devalues the earthliness of existence. For Ritter, any idea that there is something that transcends life and the world must be abandoned: Phenomenological reflection has no other referent but life, however spiritual it may be, in the body and on earth. Ritter suggests that Patočka’s theory of movements appears to present the first and second movements of acceptance and defence as referring to the body and finitude, and the third as referring to the soul and to overcoming the finite with the infinite. Although Ritter acknowledges Karfík’s reading of Patočka with respect to the absolute turning into the finite, he seeks greater clarity and a more radical drawing of conclusions: “it is not only unnecessary but mistaken to want to jump … out of one’s finite skin” (p. 174). As Ritter says elsewhere, we “must perform all the three movements of existence” (p. 123). In Ritter’s opinion, the division introduced by Patočka does not do justice to the task of existence, which consists in “being at one with oneself.” In other words, “there is no soul/self detachable from the bodily, inter-subjective, and whole-related movement of existence” (p. 124).

I applaud Ritter for attempting to present a more balanced view, one which considers finitude, the objective body, and the performance of existence in the world while not losing Patočka’s own argument concerning a-subjectivity. In this respect, Ritter confirms the intuition of contemporary phenomenology (Emmanuel Falque and others) that phenomenological reflection has its limits and brings media philosophy into play in order to enrich it. This connection, however original and oft repeated it may be, remains somewhat vague. The goal is “to disclose, and elucidate, some *objective* processes in their, paradoxically, *non-objective* conditioning of appearing” (p. 131). If I understand correctly, Ritter is suggesting that these trans-subjective conditions through which the subject realizes itself may be the concepts of cultural techniques in media philosophy. This should, Ritter argues, allow for conceiving existence in terms of the decentred subject and at the same time soften Patočka’s harsh comments on the objectification of existence, although these should be understood in their proper context. Is this a way forward in unfolding the possibilities of a-subjective phenomenology? Time, perhaps, will tell. I believe Ritter’s turn to media philosophy is potentially helpful, but it appears in rather a random section of the book, well-structured but lacking in content, and especially lacking any explanation as to how the spiritual aspect of existence—the aspect that most interests Patočka—relates to technique as the medium of existence.

The argument in the final three chapters is much more coherent. Here Ritter develops the theme of “shaken” existence in the world and offers a constructive proposal for understanding existence and living through the ontological *polemos* of being in the world. For Patočka, the world is in crisis because care for the soul, which also implies care for the polis, is replaced and even suppressed by the rule of Force (*Gestell*), that is, the objective manipulation of being, the subject, and relations. On the individual level, (self-)sacrifice breaks with this logic. Ritter is nevertheless more interested in the inter-subjective way of dealing with the crisis, namely Patočka’s ambiguous category of *the solidarity of the shaken*. One may expect Ritter to venture into political philosophy at this point, but he does not; rather, and this is to be welcomed, he patiently analyses the existential aspect: “What is it, actually, that ‘the shaken’ … have in common?” (p. 154) His answer suggests that shaken-ness is a general experience lived through the singularity of every human being. Chaos, meaninglessness, uncertainty, in a word, finitude, is always *my* finitude, although it is also a common experience between *you* and *me*. This negative affection, as Ritter calls it, shared inter-subjectively, has the potential to create the spiritual force of critical consciousness. Questions remain, however: Does Patočka’s thought offer more than warnings and prohibitions? Can shaken-ness serve as the foundation for something positive which bestows meaning?

In providing an answer, Ritter returns to Patočka’s movements of existence and argues again against their division, or, more precisely, against the elevation of the third, historical movement at the expense of the other two, which Patočka deems a-historical. For Ritter, being rooted physically in the world and defending life in its everyday needs must be taken both positively and historically. Patočka is perhaps right to suggest that we need to go through the nihil to understand “what is going on in history.” Ritter adds, however, and rightly in my opinion, that this is only the first word, not the last.

*Omnia vincit amor*, the title of the final chapter, suggests the direction Ritter will take in answering the question of how to think with Patočka beyond Patočka. Love is the way, perhaps the only viable way, to accept, defend and also transcend finite life without exiting the world and life as such: “One must seek to realize the infinite through accepting one’s own finitude” (p. 174). Love is inter-subjectivity, embodied and lived and happening between *you* and *I*. Love performs self-transcendence towards the other. Love establishes the kingdom of God among us and transcends the need to search for the “trans-human and singularly non-existent” (p. 177). Ritter suggests that ultimately Patočka falls prey to extra-terrestrial escapism, although paradoxically it is Patočka’s notion of the transubstantiation of life, of yielding unconditionally to others, which allows Ritter to argue against him. In short, Ritter is suggesting that love does not withdraw us from the world but transforms our being in the world.

Ritter’s criticism of the theology in Patočka and of using Patočka’s phenomenological philosophy for theological ends is clear. His sudden recourse to Feuerbach in the final chapter should strengthen his rethinking existence in terms of diving *into the world*. The question remains, however, is this *apologia pro philosophia—*the defence of *human, all too human*—vis-a-vis both Patočka’s thought and theological thought necessary? Is all theology looking for an extra-worldly solution to worldly—finite—existence? It seems as if there have been only bad theological responses to good philosophical questions. Who is responsible for this impasse reflected in the final pages of Ritter’s book? Theologians, for pretending to adopt the task of thinking while tacitly presupposing the answer before the questions are even asked? Or philosophers, who know about the poverty of much theology but pretend to know nothing about the kenotic tone of theological poverty? Perhaps we can, in fact, turn to Patočka for a two-fold lesson: That we must walk eternally on earth; but this does not prevent us from “touching the stars,” as Patočka writes in his very first published essay on theology and philosophy in 1929. The challenge applies both to theologians and to philosophers, who must alike embrace love as something which shows us, as Ritter touchingly writes, “that there is no greater thing than life” (p. 182).

## Footnotes

1 Pesaresi (2020).

2 Brinton (2020).

3 Koci (2020).

4 Findlay (2002).
